# Supplementary material for: Evaluating Amazon effects and the limited impact of COVID-19 with purchases crowdsourced from US consumers
Source: PLoS One. 2025 Nov 7;20(11):e0336571. doi: 10.1371/journal.pone.0336571 (PMC12594372; doi:10.1371/journal.pone.0336571)
Supplement: S1 File — (PDF) [file pone.0336571.s001.pdf]

Supporting Information

Purchases data example rows

Each row in the purchases dataset represents a purchase by a particular user and includes an order date, product code (ASIN/ISBN), product title, per-unit price and quantity, state the item was shipped to, and a category assigned by Amazon. Purchases are linked to a single user and their demographics data via a response ID. To better illustrate the dataset, we provide an example set of rows in Table S1.

Table S1. Example rows from one user's Amazon purchases data.

Order Date	Price	Quantity	State	Title	ASIN/ISBN	Category	ResponseID
2018-01-21	$23.07	1.0	OK	OTTERBOX SYMMETRY SERIES Case for iPhone 8 PLUS & iPhone 7 PLUS (ONLY) - Frustration Free Packaging - SALTWATER TAFFY (PIPELINE PINK/BLAZER BLUE)	B01K6PBRSW	CELLULAR_PHONE_CASE	R_2zARigFd
2018-02-06	$15.91	1.0	OK	Strength in Stillness: The Power of Transcendental Meditation	1501161210	ABIS_BOOK	R_2zARigFd
2018-04-03	$5.99	1.0	OK	Square Reader for magstripe (with headset jack)	B00HZYK3CO	MEMORY_CARD_READER	R_2zARigFd
2018-06-11	$4.89	1.0	OK	Dove Advanced Care Antiperspirant Deodorant Stick for Women, Original Clean	B00Q70R41U	BODY_DEODORANT	R_2zARigFd

Sample demographics

Tables S2-S4 report on the Amazon user sample demographics. We include comparisons to the US population using 2021 US census data for sex (33), age (34), and household income (35). Given the users in the Amazon sample are at least 18 years of age, we compare the sample data to census data for the 18 or older population. Users responded about their race and ethnicity via multiple choice; counts indicate whether a category was selected at all and are not expected to sum to the total N=5027.

Table S5 shows the number of survey participants from each U.S. state as well as Washing DC and Puerto Rico, in comparison to population estimates from the U.S. Census Bureau (36). Similarly to Tables S2-S4, census data are for the 18+ population in order to provide a better comparison to the survey participants, who were required to be 18+. U.S. state/territory of residence for each person in the sample was determined based on their survey response reporting state of residence in 2021.

Table S2. Sample demographics compared to 2021 US census data for sex (33), age (34), and household income (35).

	Survey		Census
**Attribute**	N	%	%
**Gender**			
Female	2589	51.5%	51%
Male	2311	46.0%	49%
Other	127	2.5%	
**Age**			
18 - 34 years	2581	51.4%	29.4%
35 - 54 years	1917	38.1%	32.3%
55 and older	529	10.5%	38.3%
**Household income**			
Less than $50,000	1874	37.3%	35.5%
$50,000 - $99,999	1824	36.3%	30.3%
$100,000 or more	1253	24.9%	34.1%
Prefer not to say	76	1.5%	

Table S3. Sample race and ethnicity.

**Race / ethnicity**	**N**
White	4133
Hispanic	549
Asian	483
Black	448
Other	263

Table S4. Sample household sizes.

**Household size**	**N**
1	1199
2	1590
3	983
4+	1255

Table S5. Sample geographic distribution by US state/territory compared to population estimates from the US census.

	survey		census
**US state/territory**	N	%	%
Alabama	67	1.3%	1.5%
Alaska	10	0.2%	0.2%
Arizona	95	1.9%	2.2%
Arkansas	46	0.9%	0.9%
California	505	10.0%	11.6%
Colorado	91	1.8%	1.8%
Connecticut	39	0.8%	1.1%
Delaware	13	0.3%	0.3%
District of Columbia	14	0.3%	0.2%
Florida	327	6.5%	6.8%
Georgia	161	3.2%	3.2%
Hawaii	21	0.4%	0.4%
Idaho	18	0.4%	0.6%
Illinois	216	4.3%	3.7%
Indiana	121	2.4%	2.0%
Iowa	46	0.9%	0.9%
Kansas	39	0.8%	0.9%
Kentucky	91	1.8%	1.3%
Louisiana	56	1.1%	1.3%
Maine	19	0.4%	0.4%
Maryland	103	2.0%	1.8%
Massachusetts	117	2.3%	2.1%
Michigan	164	3.3%	3.0%
Minnesota	97	1.9%	1.7%
Mississippi	35	0.7%	0.9%
Missouri	67	1.3%	1.8%
Montana	8	0.2%	0.3%
Nebraska	34	0.7%	0.6%
Nevada	54	1.1%	0.9%
New Hampshire	21	0.4%	0.4%
New Jersey	117	2.3%	2.8%
New Mexico	27	0.5%	0.6%
New York	300	6.0%	6.0%
North Carolina	189	3.8%	3.2%
North Dakota	5	0.1%	0.2%

Ohio	219	4.4%	3.5%
Oklahoma	60	1.2%	1.2%
Oregon	103	2.0%	1.3%
Pennsylvania	275	5.5%	3.9%
Rhode Island	18	0.4%	0.3%
South Carolina	69	1.4%	1.6%
South Dakota	11	0.2%	0.3%
Tennessee	105	2.1%	2.1%
Texas	384	7.6%	8.6%
Utah	36	0.7%	0.9%
Vermont	12	0.2%	0.2%
Virginia	148	2.9%	2.6%
Washington	123	2.4%	2.3%
West Virginia	23	0.5%	0.5%
Wisconsin	100	2.0%	1.8%
Wyoming	6	0.1%	0.2%
Puerto Rico	0	0.0%	1.0%

Census data on e-commerce retail sales and sample expenditure

Fig S1 shows quarterly e-commerce retail sales from the U.S. Census Bureau (22) from our study period extending to 2024-Q3, which is the most recently available data at the time of analysis. For comparison, we show our sample data, also aggregated to quarterly expenditure. The Pearson correlation between the sample and census expenditure data computed over our study period (2018-Q1 to 2022-Q3), is r=0.982 ($p<0.001$).

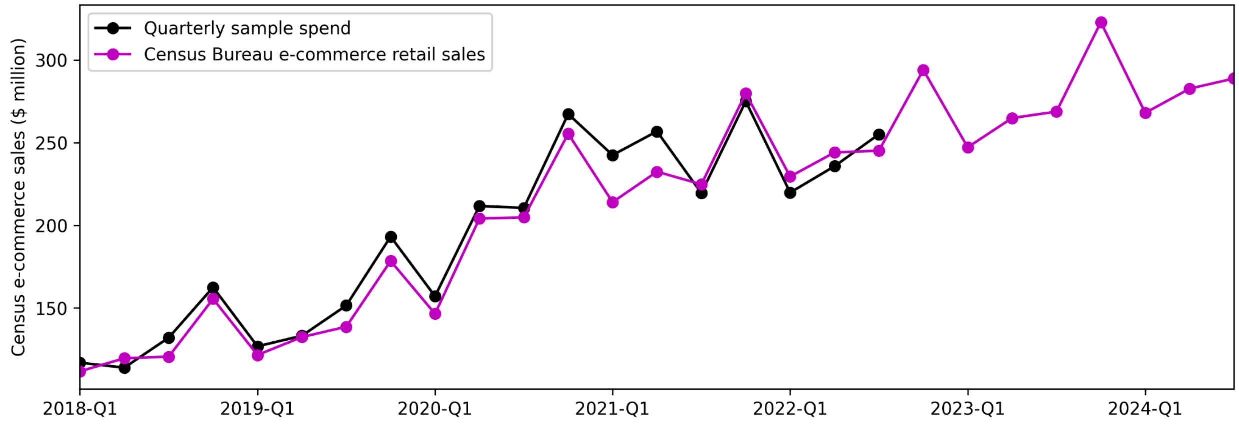

Fig S1. Quarterly e-commerce retail sales from the Census Bureau compared to the quarterly expenditure from our sample data, extending beyond our study period.

Robustness check on the role of groceries in the sample metrics

As a robustness check we test whether the patterns in the consumer purchase behavior metrics used in this paper are consistent with and without the inclusion of grocery purchases. We reproduce the monthly spend, products, and frequency time series shown in Fig 1 with and without grocery related purchases, and plot the metrics in Fig S2. We can see groceries helped drive growth in these metrics, yet the metrics are still highly correlated, showing that the patterns are relatively consistent across the purchases data with and without the grocery purchases. For sample spend: Pearson r=0.999 ($p<0.001$); for orders made: Pearson r=0.999 ($p<0.001$); for products purchased: Pearson r=0.996 ($p<0.001$).

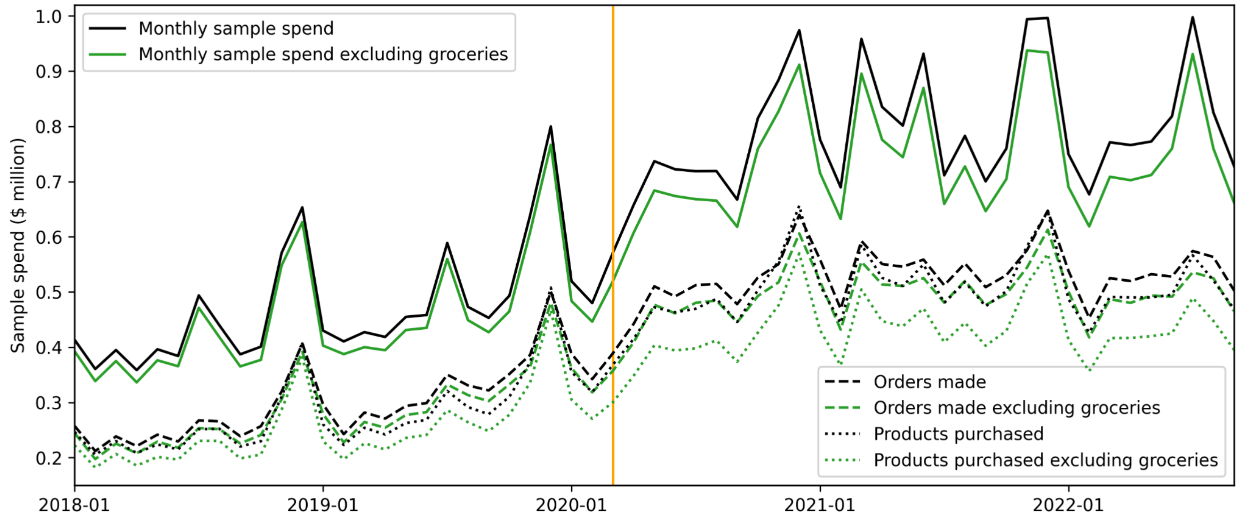

Fig S2. Purchasing metrics computed for our sample with and without grocery related products.

The impact of Amazon Prime Day on purchase behavior metrics

Amazon's major recurring sales event, "Prime Day", occurred during the following months in our data (23): 2018-07, 2019-07, 2020-10, 2021-06, 2022-07. We statistically test for the impact of Prime Day on purchase behaviors for the users in our panel data using the following OLS model:

$$y_{i,t} = intercept + \beta_1 \cdot isPrimeMonth_t + \beta_2 \cdot t + \sum_m \beta_m \cdot month_t + \beta \cdot X_i + e_i$$

Where $y_{i,t}$ is the purchasing behavior metric (distinct products/purchase days) for month t by user i. $isPrimeMonth_t$ is the variable of interest, set to 1 when the month includes Prime Day, 0 otherwise. Results are reported in Table S6, showing Prime Day significantly increased monthly purchasing metrics ($p<0.001$).

Table S6. Regression results estimating the impact of Amazon Prime Day on monthly purchasing metrics.

	**Purchase days**	**Distinct products**
**Intercept**	2.035*** (0.093)	3.832*** (0.229)
**Sex (Ref: Male)**		
Female	0.586*** (0.083)	1.810*** (0.209)
**Age (Ref: 35 - 54 yrs)**		
18 - 34 yrs	-0.881*** (0.090)	-2.198*** (0.232)
55+	-0.202 (0.140)	-0.946** (0.344)
**Income (Ref: $50k - $100k)**		
Less than $50k	-0.581*** (0.089)	-1.196*** (0.225)
$100k or more	0.954*** (0.115)	2.240*** (0.292)
**Month (Ref: 1)**		
2	-0.404*** (0.018)	-0.808*** (0.057)
3	-0.083*** (0.020)	0.019 (0.069)
4	-0.151*** (0.020)	-0.230*** (0.063)
5	-0.055** (0.020)	-0.136* (0.063)
6	-0.141*** (0.021)	-0.251*** (0.065)
7	-0.125*** (0.024)	-0.304*** (0.079)
8	-0.055** (0.020)	-0.087 (0.064)
9	-0.299*** (0.020)	-0.710*** (0.062)
10	-0.188*** (0.021)	-0.459*** (0.067)
11	0.139*** (0.023)	0.861*** (0.076)
12	0.624*** (0.024)	2.175*** (0.086)
**t**	0.039*** (0.001)	0.094*** (0.003)
**is Prime Day Month**	0.171*** (0.019)	0.733*** (0.063)
**N**	229738	229738
**R-squared**	0.087	0.067

Significance denoted as *p<0.05; **p<0.01; ***p<0.001.

Distribution of purchase behavior metrics and changes over time

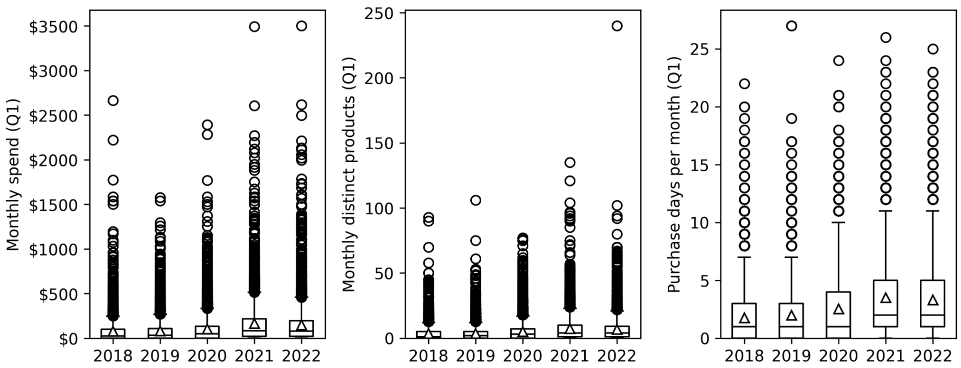

Fig S3. Fig 2 reproduced to include outliers, showing the distribution of monthly metrics across users.

Table S7. Distribution of monthly total spend per user averaged over Q1 (n=4115).

Year	2018	2019	2020	2021	2022
Mean	77.49	83.05	105.46	167.15	151.67
Std	147.56	136.55	168.91	241.57	229.89
Min	0.00	0.00	0.00	0.00	0.00
25%	0.00	0.00	0.00	19.95	18.97
50%	24.99	33.55	47.59	83.09	76.89
75%	99.08	108.00	133.98	217.10	195.41
Max	2665.77	1575.20	2391.92	3492.65	3501.27

Table S8. Distribution of monthly distinct products purchased per user averaged over Q1 (n=4115).

Year	2018	2019	2020	2021	2022
Mean	3.54	3.77	5.19	7.54	6.90
Std	5.93	5.95	7.86	10.42	10.08
Min	0	0	0	0	0
25%	0	0	0	1	1
50%	1	2	3	4	4
75%	5	5	7	10	9
Max	93	106	77	135	240

Table S9. Distribution of monthly purchase days per user averaged over Q1 (n=4115).

Year	2018	2019	2020	2021	2022
**Mean**	1.78	1.98	2.55	3.50	3.31
**Std**	2.43	2.53	3.07	3.75	3.61
**Min**	0	0	0	0	0
**25%**	0	0	0	1	1
**50%**	1	1	1	2	2
**75%**	3	3	4	5	5
**Max**	22	27	24	26	25

Relationship between purchase behavior metrics

To estimate the relationship between distinct products and purchase days each month, we estimate the following OLS regression using the panel data:

$$distinctProducts_{i,t} = \beta_1 \cdot purchaseDays_{i,t} + \beta_2 \cdot t \cdot purchaseDays_{i,t}$$

Results are shown in Table S10.

Table S10. Regression results estimating relationship between distinct products purchased and purchase days per month.

	Coef
**Purchase days**	2.1428*** (0.006)
**Purchase days x t**	0.0037*** (0.000)
**Observations**	238670
**R-squared**	0.785

Significance denoted as *p<0.05; **p<0.01; ***p<0.001.

Event study with consumer purchasing behavior metrics

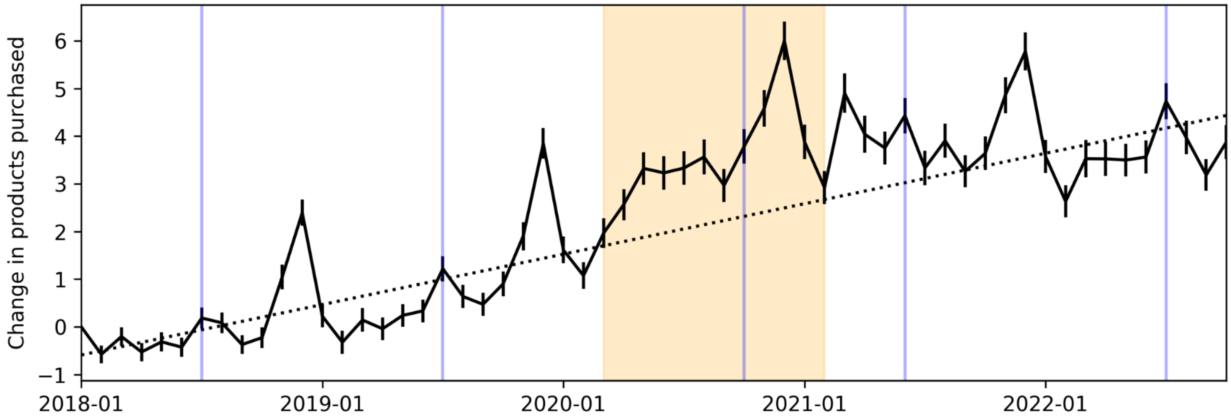

Fig S4. Graphical event study estimating change in distinct products purchased over time, using the same analysis methods as Fig 3.

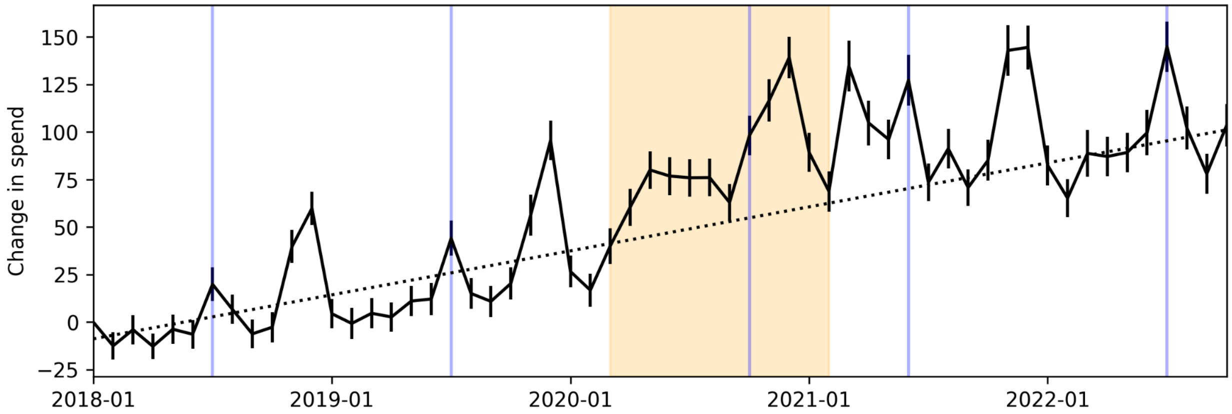

Fig S5. Graphical event study estimating change in spend ($USD) over time, using the same analysis methods as Fig 3.

Table S11. Regression results for Eq 1 estimating coefficients for the event study shown in Fig 3. The dependent variable is monthly purchase days.

Variable	Coef
Intercept	2.099*** (0.096)
**t (ref: 0)**	
t1	-0.2588*** (0.032)
t2	-0.0992** (0.034)
t3	-0.201*** (0.035)
t4	-0.0808* (0.035)

t5	-0.1497*** (0.036)
t6	0.0725 (0.038)
t7	0.0558 (0.039)
t8	-0.1086** (0.038)
t9	0.0116*** (0.04)
t10	0.3711*** (0.043)
t11	0.8664*** (0.046)
t12	0.2318*** (0.041)
t13	-0.0788 (0.041)
t14	0.158*** (0.043)
t15	0.0972* (0.044)
t16	0.2194*** (0.043)
t17	0.2562*** (0.043)
t18	0.5542*** (0.046)
t19	0.4355*** (0.045)
t20	0.3835*** (0.046)
t21	0.5549*** (0.049)
t22	0.7541*** (0.051)
t23	1.4133*** (0.054)
t24	0.7612*** (0.05)
t25	0.4961*** (0.048)
t26	0.7693*** (0.051)
t27	1.0702*** (0.056)
t28	1.4615*** (0.058)
t29	1.3638*** (0.059)
t30	1.4809*** (0.059)
t31	1.4855*** (0.06)
t32	1.2676*** (0.058)
t33	1.5451*** (0.059)
t34	1.6925*** (0.06)
t35	2.2108*** (0.062)
t36	1.7291*** (0.06)
t37	1.227*** (0.055)
t38	1.9387*** (0.064)
t39	1.692*** (0.061)
t40	1.6582*** (0.06)
t41	1.7453*** (0.06)

t42	1.4678*** (0.059)
t43	1.6854*** (0.061)
t44	1.4441*** (0.059)
t45	1.5721*** (0.06)
t46	1.8382*** (0.062)
t47	2.2583*** (0.064)
t48	1.6241*** (0.059)
t49	1.1358*** (0.054)
t50	1.5534*** (0.06)
t51	1.5175*** (0.06)
t52	1.592*** (0.059)
t53	1.5701*** (0.058)
t54	1.8276*** (0.061)
t55	1.7713*** (0.062)
t56	1.4176*** (0.057)
t57	1.6455*** (0.059)
**Sex (ref: male)**	
Female	0.5863*** (0.083)
**Age (ref: 35 - 54 years)**	
18 - 34 years	-0.8814*** (0.09)
55 years and older	-0.2021 (0.14)
**Income (ref $50,000 - $99,999)**	
$100,000 or more	0.9536*** (0.115)
Less than $50,000	-0.5812*** (0.089)
**Observations**	229738
**R-squared**	0.092

Significance denoted as *p<0.05; **p<0.01; ***p<0.001.

Table S12. Regression results for Eq 2, using coefficients from Table S11, estimating trend for the graphical event study shown in Fig 3.

	**Coef**
**Intercept**	-0.2362* (0.098)
**t**	0.0396*** (0.007)
**Observations**	26
**R-squared**	0.591

Significance denoted as *p<0.05; **p<0.01; ***p<0.001.

Relationships between demographics and purchasing behavior

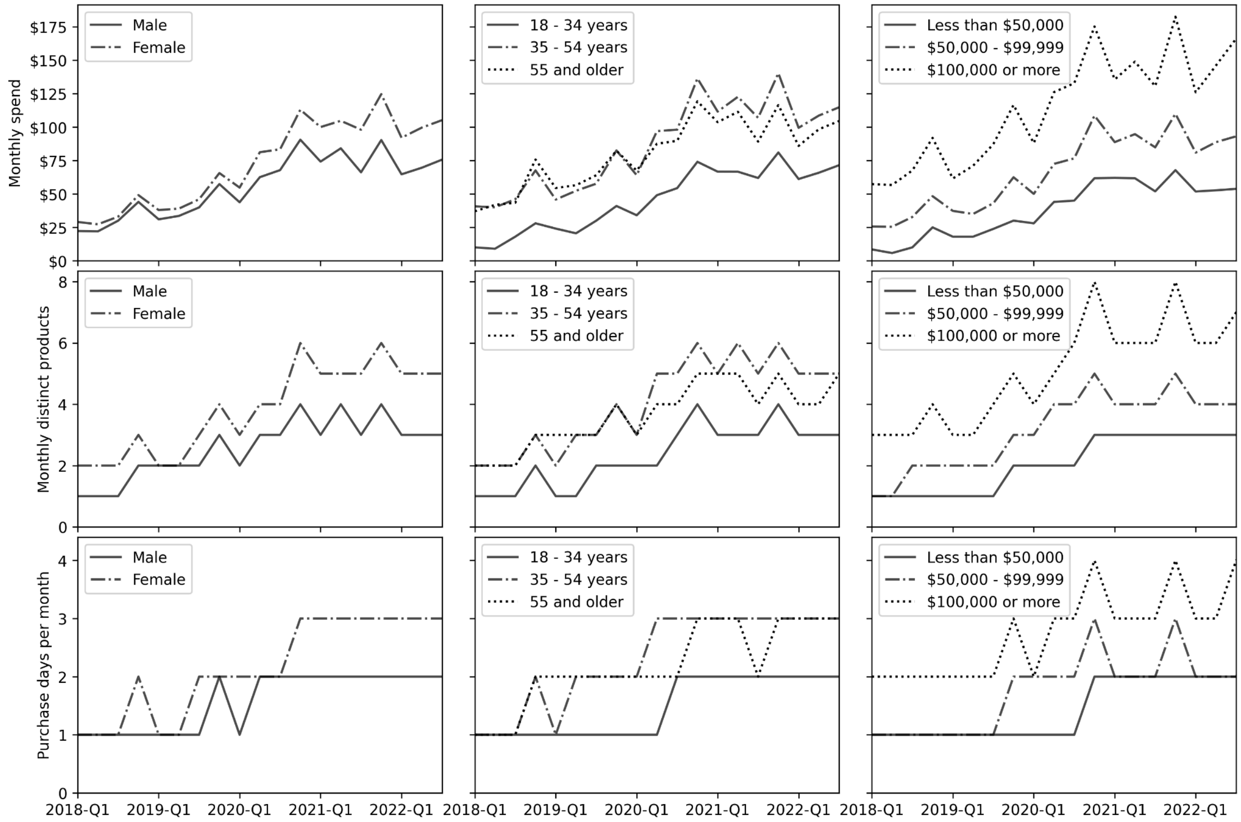

Fig S6. Variation in consumer level metrics across demographic groups, with lines showing the median computed across users in each group.

Fig S6 shows an overview of trends in purchasing behavior across demographics over time. For each of the three purchasing behavior metrics, it shows the median over the consumer-level metrics for each demographic group. This shows how female consumers in our sample spent more, bought more different products, and made purchases more often than males, and this consumption gap expanded over the study period. Younger consumers (18 - 34 years) spent less, bought fewer distinct products, and made purchases less often than older consumers. Fig S6 also compares purchasing behaviors across users' household income levels. As might be expected, spending increased with household income, with the highest income group ($100k or more) spending most and the lowest income group ($50k or less) spending least, on average. However, Fig S6 shows these expenditure differences are largely consistent with the purchasing behavior metrics (distinct products and purchase frequency), demonstrating how higher income earners' higher spending may be partly due to them buying more items more often, rather than just buying more expensive items. Fig S6 also shows differences in holiday (Q4) spending patterns. In particular, higher and medium income households show sharper spikes in purchasing metrics versus lower income households.

A limitation of these plots is that they evaluate one category of consumer demographics at a time (e.g. age) whereas these categories may be related (e.g. younger consumers may

tend to have lower household incomes). The regression analysis shown in Fig 4 (via Eq 4) controls for these demographics and their relationships.

Regression analysis

Table S13. Regression results estimating relationships between demographics and purchase frequency.

	**(1)**	**(2)**	**(3)**	**(4)**
Intercept	1.710*** (0.346)	2.338*** (0.525)	56.154 (128.563)	84.468 (47.150)
**Sex (Ref: Male)**				
Female	0.250*** (0.071)	0.819*** (0.108)	78.612** (26.239)	7.628 (9.517)
**Age (Ref: 35 - 54 yrs)**				
18 - 34 yrs	-0.647*** (0.077)	-0.807*** (0.117)	47.216 (28.652)	-11.636 (10.366)
55+	-0.049 (0.119)	-0.082 (0.181)	-21.914 (43.626)	-44.968** (15.930)
**Income (Ref: $50k - $100k)**				
Less than $50k	-0.367*** (0.085)	-0.677*** (0.129)	-7.296 (31.374)	3.402 (11.353)
$100k or more	0.665*** (0.091)	0.995*** (0.138)	-78.699* (33.417)	-22.133 (12.125)
**Race and ethnicity**				
White	0.129 (0.162)	0.468 (0.246)	54.350 (60.959)	-9.470 (21.922)
Hispanic	-0.089 (0.126)	-0.045 (0.191)	166.143*** (46.673)	9.195 (17.001)
Black	-0.397* (0.179)	-0.170 (0.272)	81.351 (67.585)	62.943** (24.171)
Asian	-0.092 (0.179)	-0.200 (0.271)	-6.167 (67.155)	-12.318 (24.193)
**Household size (Ref: 2)**				
1 (single)	-0.232* (0.098)	-0.398** (0.149)	32.877 (36.079)	26.368* (13.090)
3	0.026 (0.102)	0.145 (0.155)	20.017 (37.646)	16.108 (13.642)
4+	0.164 (0.097)	0.554*** (0.147)	79.425* (35.760)	15.126 (12.938)
**State F.E.**	Yes	Yes	Yes	Yes
**N**	3961	3961	3792	3862
**R-squared**	0.087	0.109	0.023	0.02

Significance denoted as *p<0.05; **p<0.01; ***p<0.001.

Table S13 shows regression results for Eq 4, which evaluate the relationships between consumers' demographics and purchase frequency.

Four separate OLS models were estimated, only differing in the dependent variable: (1) median monthly purchase days for 2018, (2) median monthly purchase days in 2022, (3) percent change in purchase days from 2018 to 2022, (3) percent change in purchase days from the year prior to COVID (2019-03 to 2020-02) to the period spanning the first year of COVID-19 (2020-03 to 2021-02). In addition to the demographic variables shown in Fig 4, the OLS models included fixed effects for each users' US state of residence.

Amazon acquisitions and product categories

When analyzing relationships between Amazon purchases and brick-and-mortar retail, we focus on three retail sectors in which Amazon has substantially invested: Books, shoes, and grocery. Amazon started as an online marketplace for books, describing itself as "Earth's Biggest Bookstore", and acquired other book sellers to extend its catalog (13). In 2009, Amazon acquired the leading online footwear company Zappos for $1.2 billion (14), which was the company's largest acquisition at the time (37). In 2017, Amazon purchased the grocery chain Whole Foods Market Inc. for $13.7 billion and analysts speculated the grocery stores could help Amazon expand its distribution network (15,38).

Product categories

We use the 'Category' label that Amazon assigned to each purchase in our dataset to determine whether a purchase is categorized in our analyses as 'Books', 'Shoes', or 'Grocery'.

Books purchase categories. 'ABIS_BOOK', 'BOOK', 'BOOKS_1973_AND_LATER'.
We analyze a total of 83,140 book purchases from N=4,188 users.

Shoes purchase categories. 'SHOES', 'TECHNICAL_SPORT_SHOE', 'BOOT', 'SANDAL', 'SLIPPER'.
We analyze a total of 20,885 shoes purchases from N=3,344 users.

Grocery purchase categories. 'GROCERY', 'FOOD', 'VEGETABLE', 'FRUIT', 'DAIRY_BASED_CHEESE', 'BREAD', 'POULTRY', 'DRINK_FLAVORED', 'SNACK_CHIP_AND_CRISP', 'HERB', 'MEAT', 'FRUIT_SNACK', 'SAUCE', 'NUT_AND_SEED', 'SNACK_MIX', 'WATER', 'DAIRY_BASED_CREAM', 'NOODLE', 'PUFFED_SNACK', 'PACKAGED_SOUP_AND_STEW', 'MILK_SUBSTITUTE', 'CRACKER', 'COOKIE', 'SUGAR_CANDY', 'SYRUP', 'DAIRY_BASED_BUTTER', 'BREAKFAST_CEREAL', 'COFFEE', 'TEA', 'SNACK_FOOD_BAR', 'POPCORN', 'LEAVENING_AGENT', 'Grocery', 'DAIRY_BASED_YOGURT', 'CHOCOLATE_CANDY', 'EDIBLE_OIL_VEGETABLE', 'JUICE_AND_JUICE_DRINK', 'SEASONING', 'CAKE', 'DAIRY_BASED_ICE_CREAM', 'DAIRY_BASED_DRINK', 'CONDIMENT', 'LEGUME', 'RICE_MIX', 'CHEWING_GUM', 'FISH', 'CULINARY_SALT', 'SUGAR', 'NUTRITIONAL_SUPPLEMENT', 'NUT_BUTTER', 'PASTRY',

'THICKENING_AGENT', 'CEREAL', 'BAKING_MIX', 'SALAD_DRESSING', 'HERBAL_SUPPLEMENT'.
We analyze a total of 219,749 grocery purchases from N=4,185 users.

Google mobility reports and Amazon grocery purchases

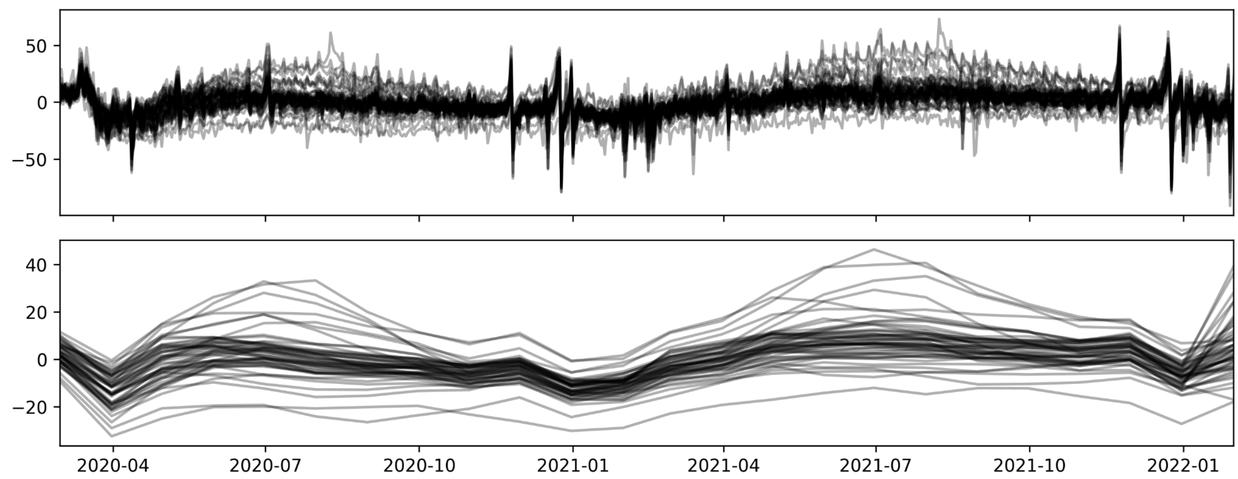

Fig S7. Google COVID-19 community mobility reports data, for each US state, (top) reported daily and (bottom) aggregated to monthly means.

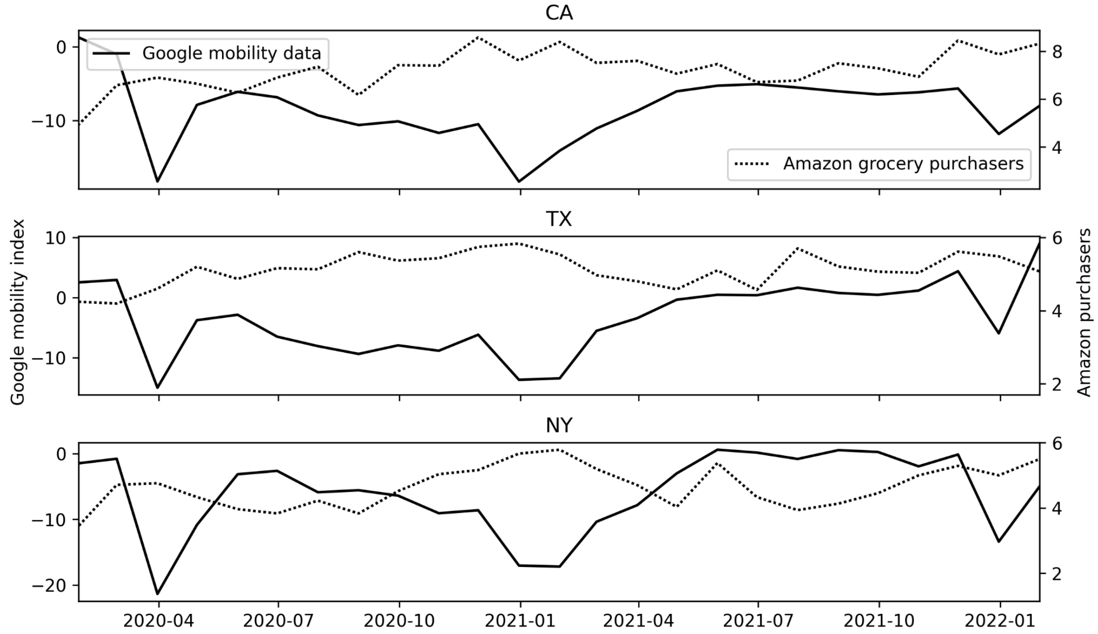

Fig S8. Monthly averages for the Google COVID-19 community mobility reports "Grocery and pharmacy" indices compared to the monthly number of Amazon users making purchases for groceries for CA (r=-0.544; p=0.007), TX (r=-0.431; p=0.040) and NY (r=-0.513; p=0.012).
