# Supplementary material for: Evaluating Amazon effects and the limited impact of COVID-19 with purchases crowdsourced from US consumers
Source: PLoS One. 2025 Nov 7;20(11):e0336571. doi: 10.1371/journal.pone.0336571 (PMC12594372; doi:10.1371/journal.pone.0336571)
Supplement: Attachment — Submitted filename: [October 2025] Response to Reviewers.pdf [file pone.0336571.s004.pdf]

Response to Reviewers

Evaluating Amazon effects and the limited impact of COVID-19 with purchases crowdsourced from US consumers
October 2025

Dear Reviewers,

Thank you again for generously taking the time to review our manuscript. Your thoughtful comments have improved this work.

Please see that we have addressed your final comments by reducing the number of words in the abstract and by updating the footnote in Table 1.
